# Progressive, Transgenerational Changes in Offspring Phenotype and Epigenotype following Nutritional Transition

**DOI:** 10.1371/journal.pone.0028282

**Published:** 2011-11-30

**Authors:** Graham C. Burdge, Samuel P. Hoile, Tobias Uller, Nicola A. Thomas, Peter D. Gluckman, Mark A. Hanson, Karen A. Lillycrop

**Affiliations:** 1 Academic Unit of Human Development and Health, Faculty of Medicine, Institute of Developmental Sciences, University of Southampton, Southampton General Hospital, Southampton, United Kingdom; 2 Department of Zoology, Edward Grey Institute, University of Oxford, Oxford, United Kingdom; 3 Liggins Institute and National Research Centre for Growth and Development, University of Auckland, Auckland, New Zealand; 4 Faculty of Natural and Environmental Sciences, Institute of Developmental Sciences, University of Southampton, Southampton General Hospital, Southampton, United Kingdom; Ludwig-Maximilians-Universität München, Germany

## Abstract

Induction of altered phenotypes during development in response to environmental input involves epigenetic changes. Phenotypic traits can be passed between generations by a variety of mechanisms, including direct transmission of epigenetic states or by induction of epigenetic marks *de novo* in each generation. To distinguish between these possibilities we measured epigenetic marks over four generations in rats exposed to a sustained environmental challenge. Dietary energy was increased by 25% at conception in F0 female rats and maintained at this level to generation F3. F0 dams showed higher pregnancy weight gain, but lower weight gain and food intake during lactation than F1 and F2 dams. On gestational day 8, fasting plasma glucose concentration was higher and β-hydroxybutyrate lower in F0 and F1 dams than F2 dams. This was accompanied by decreased phosphoenolpyruvate carboxykinase (PEPCK) and increased PPARα and carnitine palmitoyl transferase-1 mRNA expression. PEPCK mRNA expression was inversely related to the methylation of specific CpG dinucleotides in its promoter. DNA methyltransferase (Dnmt) 3a2, but not Dnmt1 or Dnmt3b, expression increased and methylation of its promoter decreased from F1 to F3 generations. These data suggest that the regulation of energy metabolism during pregnancy and lactation within a generation is influenced by the maternal phenotype in the preceding generation and the environment during the current pregnancy. The transgenerational effects on phenotype were associated with altered DNA methylation of specific genes in a manner consistent with induction *de novo* of epigenetic marks in each generation.

## Introduction

Organisms respond to changes in their environment in a variety of ways, both adaptive and non-adaptive [1]. Over longer time scales, natural selection on heritable genetic variation may enable populations to become adapted to what was originally a novel environment. In the shorter term, plastic responses to environmental conditions can enhance fitness. These mechanisms also have the advantage that they are potentially reversible [2], which may be advantageous if the environmental change is not sustained. In mammals the signals that induce developmental plasticity are often mediated by the parents, particularly mother during fetal and neonatal life [3]–[7]. Although such parental effects need not be adaptive, by allowing structural changes to be passed on to future generations they may also facilitate the persistence of populations in stressful environments and affect the potential for natural selection [3], [6], [8]–[11]. Parental effects are likely to be particularly important in mammals as the sustained, intimate relationship between mothers and offspring during gestation and suckling facilitates transference of environmentally induced variation between generations [5], [7], [12].

There is particular interest in the way in which socioeconomic change in humans, such as increasing affluence or migration, can produce mismatch between the environment experienced by the fetus or infant, including that based on the maternal phenotype, and the actual future environment, and how this increases risk of non-communicable disease [13]. In this context, changes in nutrition between generations are of particular importance [13]. Such environmental changes usually persist over several successive generations. Whether intergenerational influences magnify or attenuate phenotypic changes in the course of a few generations is amenable to empirical testing. For example, the effects in one generation can potentially induce new maternal effects when offspring themselves are pregnant or suckling, which may lead to a more or less gradual phenotypic change across generations. However, existing animal models developed to explore the mechanisms underlying the effects of a nutritional transition have largely imposed a dietary change only on a single generation, usually the mother before or during pregnancy and/or suckling [14]. There is little experimental information in mammals about the effects of persistent environmental shifts on the phenotypes of successive generations.

Induction of altered phenotypes in the offspring by maternal effects can involve changes in the epigenome [13], [15] through altered DNA methyltransferase (Dnmt) activity and histone structure [16]. In insects and plants, regulation of developmental plasticity can involve heat-shock protein (Hsp)-90 acting via epigenetic mechanisms which buffers phenotypic change during development [17], [18]. Thus reduction in Hsp90 expression in *Drosophila* allows expression of novel phenotypes [19].

The passage of induced phenotypes between generations can also involve transmission of induced epigenetic change. For example, hypermethylation of the hepatic PPARα and glucocorticoid receptor (GR) promoters has been reported in both F1 and F2 offspring of F0 rats fed a protein restricted diet during pregnancy even though F1 dams were nourished adequately during their pregnancy [20]. Thus, stability of induced epigenetic marks across generations might occur by two possible mechanisms. One involves the transmission of induced phenotype via epigenotype through the germ line, although such epigenetic marks would need to be preserved despite demethylation of about 80% of the genome which occurs after fertilisation [21]. Alternatively, the epigenotype, and thus the phenotype, may be induced *de novo* in each generation through interactions between the maternal phenotype and the environment during her pregnancy. In addition, because the genetic material of the germ cells which will form the F2 generation develops in the F1 during F0 pregnancy [22] a stimulus operating only during that pregnancy could induce effects manifest in F2, but not F3 offspring, and this has been reported for the effects of exogenous glucocorticoid administration [23]. The two fundamental processes referred to above are analogous to those described by Crews ‘context dependent’ versus germ-line dependent'[24] or Guerrero-Bosagna and Skinner ‘intrinsic’ versus ‘extrinsic’[25].

**Figure 1:**
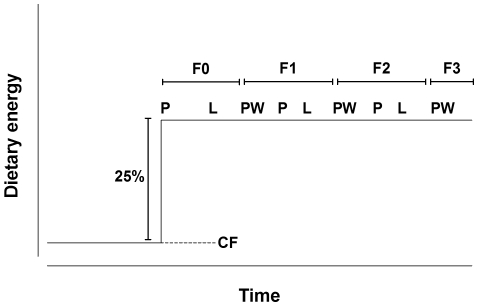
Experimental design. After conception in F0 dams, the energy content of the diet was increased by approximately 25% compared to the chow diet fed in the breeding colony. The energy content of the diet did not differ between generations during pregnancy (P), lactation (L) and to the offspring after weaning (PW).

In the present study we investigated the effect of a sustained dietary change on the induced phenotype and associated epigenetic marks of female rats over four generations. Furthermore, we attempted to distinguish whether such changes were due to transmission of induced phenotype via stable changes in epigenotype or induction of phenotypes *de novo* in each generation through interactions between the maternal phenotype and the environment during her pregnancy. We hypothesised that epigenetic marks which were transmitted directly would remain unchanged between generations, while those which were induced *de novo* in each generation would differ between generations. To mimic transition between stable environments relevant to human dietary transitions, we increased dietary energy content in the treatment group by 25% and maintained this level for the three subsequent generations, comparing the offspring to a reference group which had been fed on standard chow in the breeding colony for more than ten generations (Figure 1).

We show that despite continued exposure to a high level of dietary energy, there was a progressive shift towards improved energy balance between F0 and F2 pregnancies. This was accompanied by changes between generations in pregnant dams in the regulation of the transcription of key genes involved in hepatic lipid and carbohydrate metabolism. These were accompanied by altered patterns of DNA methylation which were associated with altered gene expression, and by epigenetic regulation of DNA methyltransferases. Furthermore, increased phenotypic and epigenetic variation was associated with decreased heat shock protein (HSP) 90 expression in gastrulating embryos in each generation. Together, our findings support the suggestion that transgenerational effects involves induction *de novo* of altered epigenetic marks in each generation and that such phenotypic changes are driven by interactions between the maternal phenotype and her environment, leading to changes in the developmental context of offspring in each generation.

## Results

**Figure 2:**
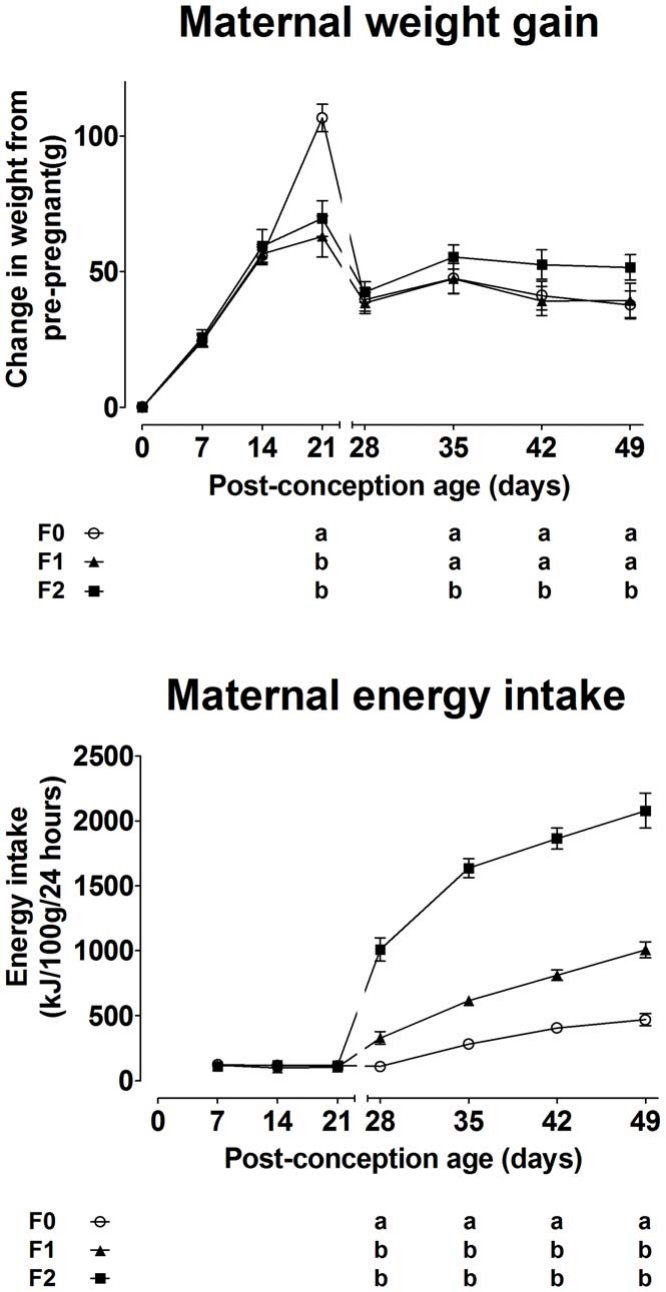
Change in maternal body weight from conception and energy intake during pregnancy and lactation. Values are mean ± SD for n = 5−7 rats per group. Different letters indicate significantly different (P<0.05) values between generations.

**Figure 3:**
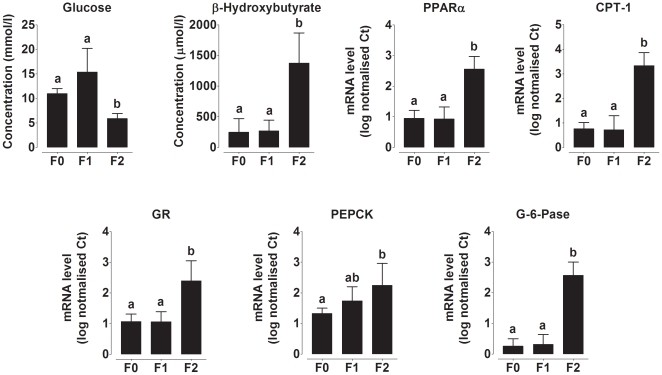
Maternal blood metabolite concentrations and mRNA expression of genes involved in hepatic gluconeogenesis and ketogenesis. Plasma fasting glucose and β-hydroxybutyrate concentrations. Hepatic PPARα, carnitine palmitoyltransferase-1 (CPT-1), glucocorticoid receptor (GR), phosphoenolpyruvate carboxykinase (PEPCK), (G) glucose-6-phosphatase (G-6-Pase) mRNA expression. Values are mean ± SD for n = 5−7 rats per group. Values with different letters are significantly different (P<0.05).

F0 dams gained approximately 40 g more weight at term compared to F1 and F2 dams (generation* gestational age F(184,50) 3.7, P<0.0001) (Figure 2). However, there was no difference in food intake during pregnancy between generations (Figure 2) which suggests that the increase in dietary energy provision altered nutrient partitioning. Pregnant mammals produce an exaggerated metabolic response to the additional stress of fasting, characterised by a faster induction and greater level of gluconeogenic and ketogenic activity than in non-pregnant females [26]. We therefore used the metabolic challenge of fasting to assess the metabolic phenotype of the dams on day 8 pregnancy in each generation. Plasma glucose concentration was lower (generation F(1856,5.3) 25.6, P<0.0001) and β-hydroxybutyrate (βHB) (generation F(1934712,164232) 11.8, P<0.0001) concentration was higher in F2 dams than F0 dams during this test suggesting that there was a transition towards greater glucose utilisation and increased glucose sparing by ketogenesis in F2 dams (Figure 3). These altered responses to fasting were associated with changes in mRNA expression of hepatic genes involved in gluconeogenesis and ketogenesis (Figure 3). GR (generation F(3.9,0.2) 16.5, P<0.0001), PPARα (F(5.9,0.1) 43.8, P<0.0001), carnitine palmitoy1 transferase (CPT)-1 (F(15.1,0.2) 65.1, P<0.0001), glucose-6-phosphatase (G6Pase) (F(12.1,0.1) 97.4, P<0.0001) and phosphoenolpyruvate carboxykinase (PEPCK) (F(1.3,0.3) 4.3, P = 0.001) mRNA expression was increased in F2 compared to F0 and F1 dams. These observations suggest that altered regulation of specific genes underlies phenotypic change between generations.

Weight gain post-partum (post-partum age* generation F(852,427) 2.0, P<0.0001) and food intake (post-partum age* generation F(87,44) 2.0, P = 0.009) was greater in F1 and F2 dams than F0 (Figure 2). There was no significant difference between groups in length of gestation, litter size or litter weight during suckling (data not shown). These observations show that transition between two levels of dietary energy induced changes in the phenotype of pregnant and lactating dams across generations.

**Figure 4:**
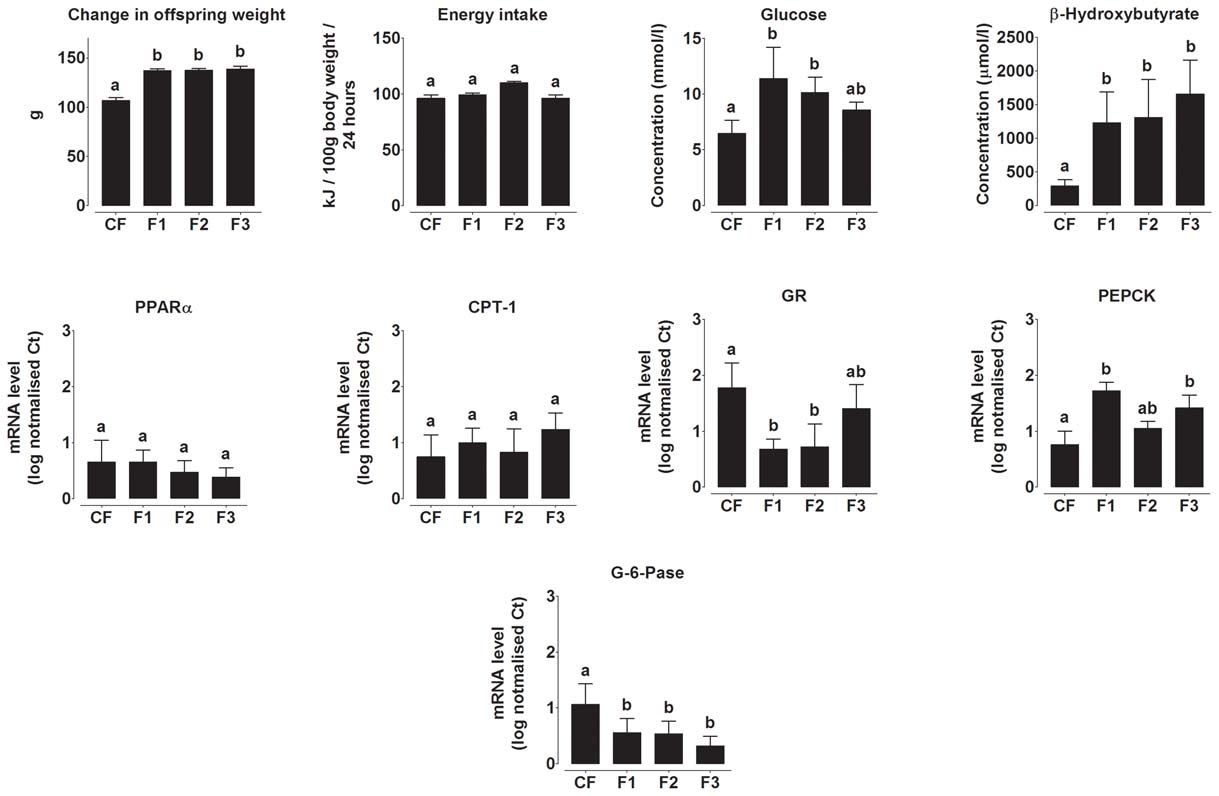
Offspring phenotype and mRNA expression of genes involved in hepatic gluconeogenesis and ketogenesis. Change in offspring body weight on day 70 compared to weaning, offspring energy intake on day 70, fasting glucose and β-hydroxybutyrate concentrations on postnatal day 70. Hepatic PPARα, carnitine palmitoyltransferase-1 (CPT-1), glucocorticoid receptor (GR), phosphoenolpyruvate carboxykinase (PEPCK), (I) glucose-6-phosphatase (G-6-Pase) mRNA expression. Values are mean ± SD for n = 5−7 rats per group. Values with different letters are significantly different (P<0.05).

The effect of increased energy intake on the phenotype of the adult offspring was assessed by comparison with the offspring of dams fed a lower energy chow diet and which were themselves fed chow from weaning (CF group). Weight gain (F(8274,1426) 5.8, P = 0.006) on postnatal day 70 was significantly greater in, but did not differ between, F1, F2 and F3 offspring of dams fed the higher energy diet than CF offspring (Figure 4). Energy intake did not differ between groups (Figure 4). Plasma glucose concentration during fasting was higher in F1 and F2 offspring, but was not significantly different from CF offspring in the F3 generation (F(24,10) 2.4, P<0.0001), while βHB concentration was higher in F1, F2 and F3 compared to CF offspring (F(2944,2220) 1.3, P = 0.027) (Figure 4). PEPCK mRNA expression was higher (F(102,3.1) 33.2, P = 0.001) and G6Pase lower (F(166,47) 3.5, P = 0.001) in all three generations compared to CF offspring (Figure 4 E to I). GR expression was lower in F1 and F2 offspring compared to CF and F3 offspring (P<0.0001). There were no significant differences between generations in PPARα or CPT-1 mRNA expression. Thus the shift in energy intake induced adjustments in the phenotype of the adult offspring which, at least in part, was reflected in altered gene expression. However, such effects were more modest than observed in pregnancy which suggests the effects of the developmental environment on the female offspring were cryptic unless challenged by the metabolic demands of pregnancy.

**Figure 5:**
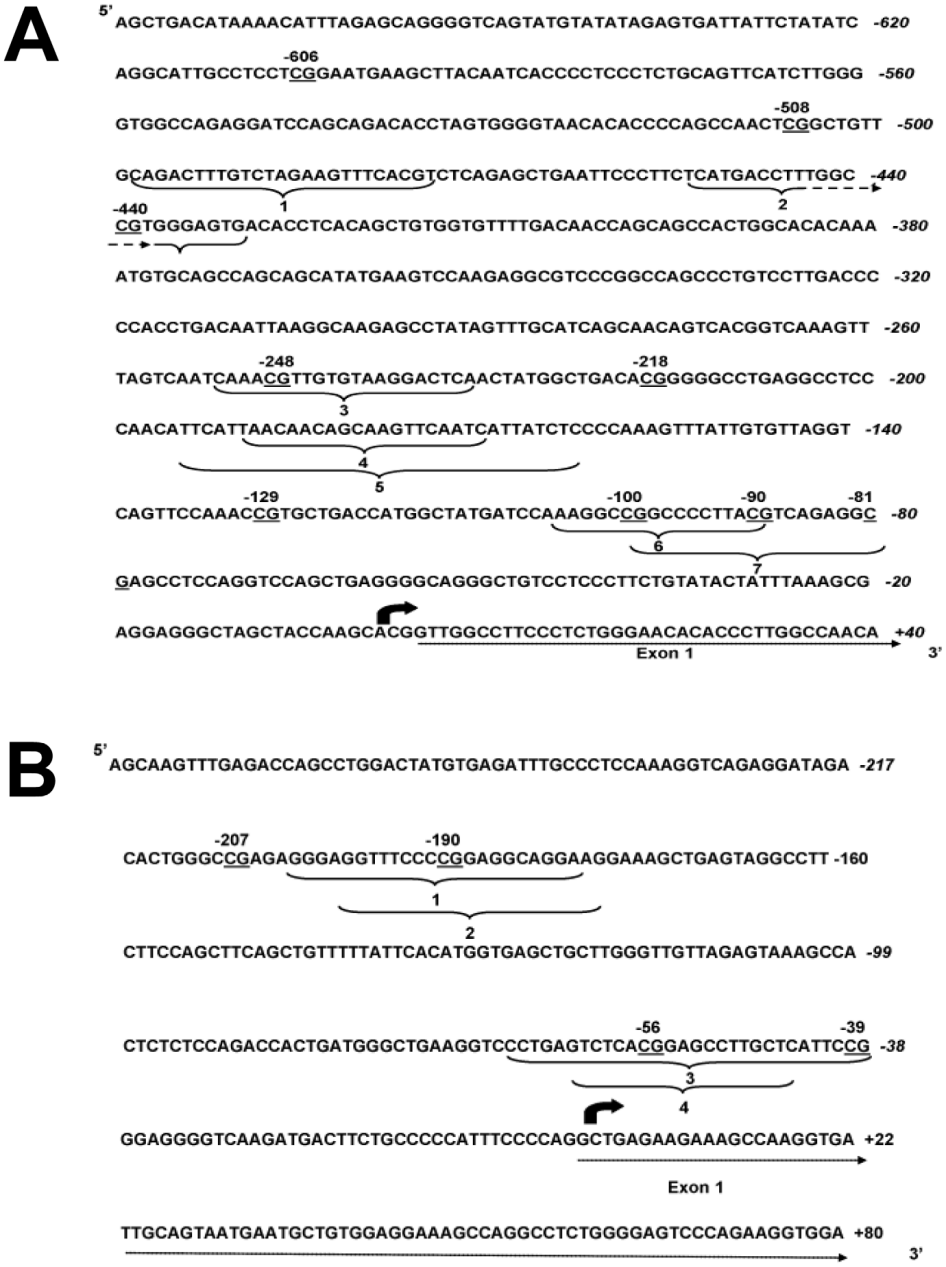
Structure of the phosphoenolpyruvate carboxykinase and DNA methyltransferase 3a promoters. Genomic sequence of the region of the (A) phosphoenolpyruvate carboxykinase promoter analysed for CpG methylation. CpG reported in the methylation analysis are underlined. Known transcription factor response elements are indicated by curved brackets; (1), heat shock factor, (2) PPAR, (3) CATT enhancer-binding protein, (4), glucocorticoid receptor (5) hepatic nuclear factor-1, (6), Krueppel-like transcription factors, (7) cAMP-response element [38]. (B)Genomic sequence of the region of the DNA methyltransferase 3a2 promoter analysed for CpG methylation. CpG reported in the methylation analysis are underlined. Putative transcription factor response elements are indicated by curved brackets; (1) nuclear factor of activated T cells, (2) retinol x receptor, (3) neurone-restrictive silencer factor, (4) mouse Krueppel factor.

**Figure 6:**
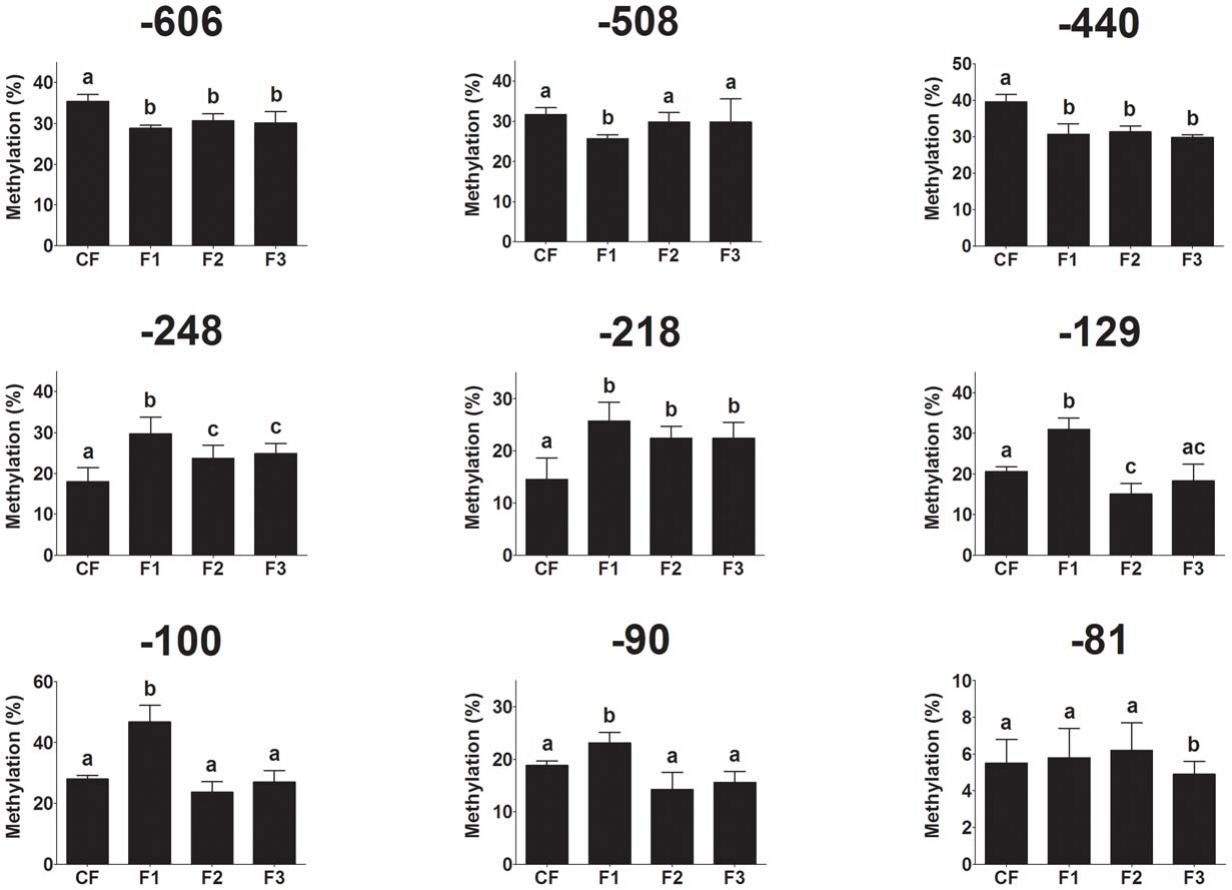
Methylation of individual CpGs in the PEPCK promoter in the liver of the adult offspring. CpG locations (bp) are relative to the transcription start site. Values are mean ± SD of 5–7 samples. Values with different letters are significantly different (P<0.05).

Because PEPCK is rate limiting in gluconeogenesis and hence is critical to fasting glucose metabolism, the mechanism underlying the change in gene expression between generations was investigated by measuring the methylation of nine individual CpGs in the PEPCK promoter (Figure 5). Compared to CF offspring, CpGs -606, -440 were hypomethylated and CpGs -248 and -218 were hypermethylated in all three generations (F(661,11) 63.7; F(1424,18) 78; F(212,7) 32.4; F(44,2) 25, respectively, all P<0.001) (Figure 6). CpGs −508, −100, and −90 were hypomethylated in F1 only (F(313,21) 15; F(1151,18) 62.5; F976,8) 9.7; F(30,7) 4.4, respectively, all P<0.05) (Figure 6). CpG -129 was hypermethylated in F1, but was hypomethylated in F2 offspring (F(458,28) 16.3, P = 0.0007) (Figure 6). CpG -81 was only hypomethylated in F3 offspring (F(257,8) 32.1, P = 0.0004) (Figure 6). The methylation status of CpGs -508 (r =  −0.521, P = 0.02), -129 (r =  −0.343, P = 0.001) and CpG -100 (r =  −0.579, P = 0.002) was significantly associated with PEPCK mRNA expression. These findings show that persistent change in dietary energy induces adjustment of the level of methylation of specific CpGs over three generations, providing a mechanism by which the effects of the developmental environment induce changes in the offspring phenotype, even though these may not become apparent until a further challenge such as pregnancy.

**Figure 7:**
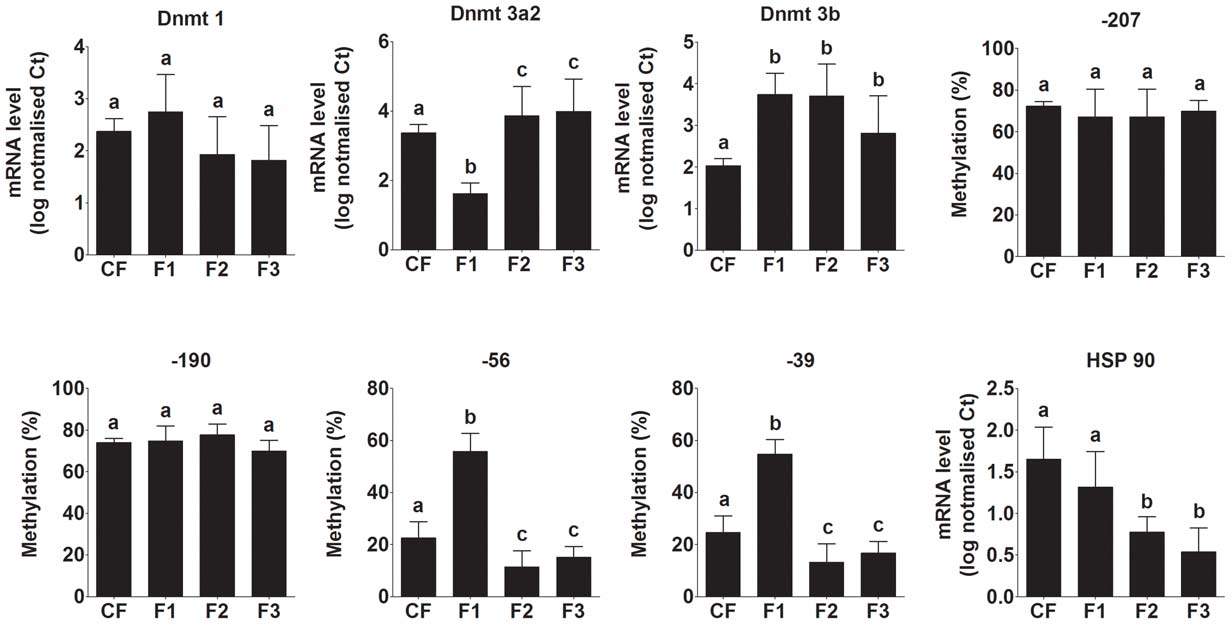
Hepatic DNA methyltransferase expression and Dnmt3a2 promoter methylation, and embryo heat shock protein 90 expression. Dnmt 1, Dnmt 3a2 and Dnmt3b mRNA expression in the liver of non-pregnant adult offspring on postnatal day 70. Methylation of individual CpGs in the Dnmt 3a2 promoter in adult offspring. HPS 90 mRNA expression in post-conception day 8 gastrulating embryos. Values are mean ± SD, n = 5 to 7 samples per group. Values with different letters are significantly different (P<0.05).

The mRNA expression of Dnmt1, 3a and 3b was measured in the liver of both adult non-pregnant and pregnant offspring (Figure 7). Dnmt1 expression did not differ significantly between groups. However, expression of Dnmt3a was decreased in F1 non-pregnant offspring, but increased in F2 and F3 offspring (F(726,43) 40.1, P<0.0001) compared to CF offspring. Dnmt3b mRNA expression was increased compared to CF offspring in all three generations (F(562,46) 12.2,P = 0.0001). These findings indicate that overall capacity to induce DNA methylation *de novo* differed between generations and thus suggests a mechanism for altered epigenetic regulation. Because Dnmt3a showed marked variation in expression between generations, we investigated the mechanism underlying changes in Dnmt3a expression we measured the methylation status of four CpGs in the Dnmt3a2 promoter which accounts of approximately 50% of the Dnmt3a expression in adult liver and is the predominant isoform in developing tissues [27]. The methylation of CpGs -207 and -190 was not altered by generation or pregnancy (Figure 7). However, the methylation of CpGs -56 and -39 was increased in F1 non-pregnant offspring, but decreased in F2 and F3 offspring (Figure 7). Both of these CpGs were hypermethylated in F1 and F2 pregnant offspring compared to their non-pregnant siblings. These observations suggest that altered epigenetic regulation of Dnmts is involved in phenotypic variation between generations.

Finally, because HSP90 has been implicated in regulating developmental plasticity via a mechanism involving epigenetic change [17], we measured the mRNA expression of HSP90β in day 8 gastrulating embryos. HSP90 mRNA expression differed significantly between generations (F(770,13) 59.2, *P*<0.0001). There was a non-significant trend (P<0.1) towards lower HSP90 expression in F1 than CF embryos. HSP90 expression was significantly lower in F2 and F3 embryos than in CF and F1 embryos (Figure 7) which suggests less stringent regulation of development and thus increased plasticity [19].

## Discussion

We show that a sustained change in energy intake, starting when the F1 generation was conceived, induced progressive changes in capacity to regulate glucose and fatty acid metabolism in the offspring. This led to improved capacity to maintain glucose and lipid homeostasis during fasting, and hence to meet the demands of maternal and fetal tissues for these nutrients during pregnancy, by the F2 generation. Together with progressive changes between generations in the regulation of body weight and food intake when pregnant, these findings suggest phenotypic adjustments in response to the prevailing availability of nutrients. Such effects are consistent with the suggestion that cues during development lead to adjustments in developmental pathways to attributes that may prove beneficial to the organism [2]; while this concept originally related to effects on morphology, we have extended it to homeostatic settings. It may be argued that the nutritional demands of pregnant and developing animals reflect tissue functions which are the products of natural selection. If so, capacity to undergo adjustments in metabolic pathways to meet such demands when faced with an environmental nutritional challenge may confer future adaptive advantage to the dam and offspring. The induction of phenotypes in the offspring is driven by interaction between the phenotype of the mother, induced during her own development, and the prevailing environment during pregnancy and lactation. Thus differences in the phenotype of the mother, and hence her interaction with the environment, in each generation may modify the developmental context to which the offspring is exposed, and so provide a mechanism for phenotypic shift over several generations when a population is presented with a sustained environmental change.

The induction of phenotypic changes across generations involved changes in transcription of specific genes associated with differential methylation of individual CpGs in each generation. We show that a shift in energy intake during F0 pregnancy induced differences in the pattern of methylation of individual CpGs in the PEPCK and Dnmt 3a2 promoters in the F3 generation. Such epigenetic changes in F3 suggest either transmission of epigenetic marks unchanged through preceding generations, which would need to be induced in germ cells and preserved through genome-wide demethylation following fertilisation [28], or alternatively the induction of epigenetic marks *de novo* during F3 development. We have shown previously that epigenetic changes can indeed be transmitted apparently unchanged between generations [20], although in our previous study we did not employ a sustained dietary change beyond F0 pregnancy. In addition, the technique we used in the previous study did not allow us to detect changes in methylation at specific CpGs.

In the present study, the level of methylation of specific CpGs differed between sequential generations. We assumed that epigenetic marks which were transmitted directly would not change significantly between generations, while those which were induced *de novo* in each generation would show variation between generations. While it is possible that epigenetic marks which were induced *de novo* in each generation may be similar between generations, the converse is unlikely to be true. Therefore, the present findings suggest that changes in DNA methylation between generations resulted from induction of methylation patterns *de novo* in each generation. Since changes in epigenetic marks were associated with altered mRNA expression of specific genes, they are consistent with progressive adjustment of offspring phenotype between generations as a result the interaction between the phenotype of the pregnant dam and the prevailing environment. However, because the epigenetic changes are induced at least in part *de novo*, the phenotype of the offspring will be more able to be adjusted to new environmental information than if the pattern of DNA methylation had been transmitted unchanged from mother to offspring. Presumably the pattern of epigenetic change will eventually become stable if the environment does not change. Our data suggest that this has not occurred by F3 in the rat using this particular environmental shift, but this warrants further investigation. It has been reported previously that impaired glucose homeostasis induced in the offspring by transient maternal protein restriction in F0 can be passed to F3 [29]. However, we did not find evidence for a substantial contribution of such grand maternal effects to the phenotype of F3 offspring in this study. One possible explanation is that the nature of the environmental challenge is an important factor for determining the pattern of phenotypic and epigenetic changes between subsequent generations.

DNA methylation is regulated through the combined activities of Dnmts acting over the course of development to induce patterns of gene expression specific to individual cell types and to maintain the epigenome in post-mitotic cells [21]. Thus modulation of Dnmt1, 3a2 and 3b expression reflects changes in capacity to induce and maintain epigenetic marks throughout the life course and so provides one mechanism for induced epigenetic and phenotypic change between generations. Furthermore, at least for Dnmt3a2 changes in expression between generations were negatively associated with altered methylation of its promoter. Altered expression of Dnmts might be expected to lead to genome wide changes in DNA methylation and gene expression. However, there is increasing evidence that the Dnmts are targeted to specific genes via histone deacetylases, histone methyltransferases and specific transcription factors [30], [31] and so provides a mechanism for variation in the methylation of specific CpGs such as observed in PEPCK.

**Figure 8:**
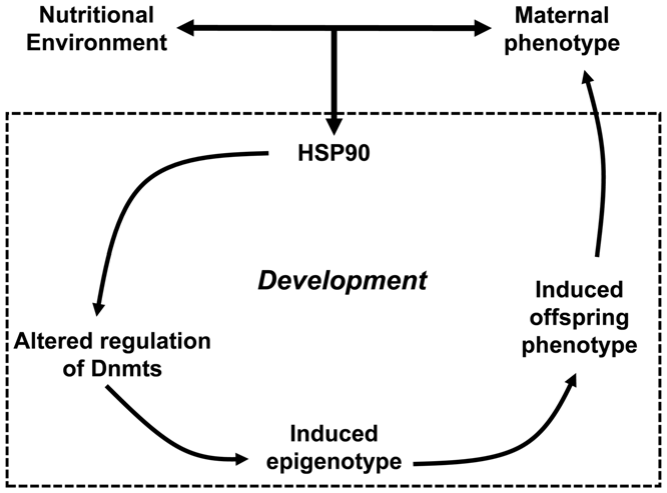
Scheme for induction of phenotypic and epigenetic variation between generations.

Traditionally, lower HSP90 expression might be viewed as a reduction in canalisation, allowing development to be less constrained and more responsive to external input [19], [32]. The lower HSP90 expression we observed in developing embryos may be affected by the interaction of maternal phenotype with the environment. HSP90 regulates developmental plasticity, at least in part, via epigenetic mechanisms [17], [18]. Thus together these findings suggest a pathway by which the interaction between maternal phenotype during pregnancy and the prevailing environment in each generation induces developmental cues which act through HSP90 expression and epigenetic processes to produce progressive phenotypic adjustments between generations (Figure 8).

Overall, the pattern of changes in growth and metabolism between generations provides a non-genomic mechanism by which adjustments in metabolic processes may facilitate adaptation to novel nutritional environments [2]. The directional shift in phenotype between generations could be an emergent feature of phenotypic accommodation of the novel diet in the maternal generation and the species-typical mechanisms of maternal-fetal interactions [2], [6]. Alternatively, the mechanisms involved in the transgenerational modification of homeostatic set-points may have been selected as a channel for transmission of information about diet availability across generations in the form of maternal effects [7]. Such epigenetic processes may also contribute in the longer-term to adaptation by increasing phenotypic variation which may lead to positive effects on the direction of phenotypic change (towards greater fitness) and the recurrence of induced phenotypes available to natural selection [6], [9], [33], [34]. Such processes may have important implications for the survival prospects of species facing challenges such as climate change [35] or for the consequences of migration and socioeconomic improvement on human health in subsequent generations [13].

## Methods

### Ethical statement

The study was carried out in accordance with the United Kingdom Home Office Animals (Scientific Procedures) Act (1986) and was conducted under Home Office Licence number 70–6457. The study received institutional approval from the University of Southampton Biomedical Research Facility Research Ethics Committee.

### Animals and tissues

Female Wistar rats (about 220 g) obtained from a breeding colony were maintained on standard chow for 14 days and then mated. No male was mated with any of its progeny. All diets were obtained from Test Diet (Division of Land O'Lakes Purina Feed, Richmond, Indiana, USA). F0 Dams were fed a diet containing 25% more energy compared to the breeding colony diet from conception and throughout pregnancy (n = 28 per dietary group) (Table 1). Dams were fed a diet with a similar energy content during lactation (AIN93G) and offspring were weaned onto AIN93M on postnatal day 28 (Table 1). Litters were standardised to 8 offspring within 24 hours of birth, with a bias towards females to ensure sufficient stock for mating. On postnatal day 70, F1 and F2 females were either mated or killed and tissues removed. Those which were mated were fed the same diets during pregnancy and lactation as F0 dams, and their offspring were weaned onto AIN93M. 7 Dams per generation group were killed on pregnancy day 8 and embryos and maternal tissues collected. All tissues and embryos were frozen immediately in liquid nitrogen. Blood was collected into heparinised tubes, plasma was separated by centrifugation and stored at −80^°^C. Dams and offspring were weighed and 24 hour food intake recorded at 7 day intervals.

**Table 1:** Diet composition.

	Breeding colony chow diet (2018S)	Pregnancy diet	Lactation diet(AIN-93G)	Maintenance diet(AIN-93M)
Casein (g/kg)	188	183	200	140
Cornstarch (g/kg)	450	420	397	466
Sucrose (g/kg)	50	213	100	100
Choline (g/kg)	1.1	2.8	2.5	2.5
Methionine (g/kg)	4.3	9.7	5.2	3.6
Crude fibre (g/kg)	38	50	50	50
Oil (g/kg)	60	100	70	40
Total metabolisable energy (MJ/kg)	13.7	17.2	16.4	15.78

A group of day 70 female offspring from dams in the breeding colony where female rats had been fed chow diet (2018S) (Table 1) over at least ten generations (CF offspring). Tissues were collected and stored in the same manner as offspring in the transgeneration study and used a reference for some outcomes. In addition, embryos were collected on day 8 from female rats fed the chow diet (2018S).

### Measurements of metabolites in blood

βHB and glucose concentrations in plasma were measured as described using a Konelab 20 [36].

### Real time RTPCR

Real time RTPCR was carried out essentially as described [37]. mRNA expression of hepatic genes was measured by real-time PCR. Briefly, total RNA was isolated from cells with TRIzol reagent (Invitrogen, Paisley, Scotland, U.K.), and 1 µg was used as a template to prepare cDNA with 100 units of Moloney murine leukemia virus reverse transcriptase. cDNA was amplified with real-time PCR primers (Table 2). The reaction was performed in a total volume of 25 µl with SYBR Green Jumpstart Ready Mix (Sigma, Poole, Dorset, U.K.) as described by the manufacturer. Samples were analysed in duplicate, and Ct values were normalized to cyclophilin [37].

**Table 2:** Real time RTPCR primers.

	Real time RTPCR	
	Forward Primer (5′<$>\scale 80%\raster="rg1"<$>3′)Reverse Primer (3′<$>\scale 80%\raster="rg1"<$>5′)
Gene	mRNA expression	
Cyclophilin	TTGGGTCGCGTCTGCTTCGA	GCCAGGACCTGTATGCTTCA
PPAR α	CGGGTCATACTCGCAGGAAAG	TGGCAGCAGTGGAAGAATCG
CPT-1	ACCACTGGCCGAATGTCAAG	AGCGAGTAGCGCATGGTCAT
GR1_10_	TGACTTCCTTCTCCGTGACA	GGAGCCTCCTCTGCTGCTTG
PEPCK	AGCTGCATAATGGTCTGG	GAACCTGGCGTTGAATGC
G-6-Pase	QuantiTect primer assay QT00190610	
HSP90	QuantiTect Primer Assay QT01786610	
Dnmt 1	QuantiTect Primer Assay QT00493577	
Dnmt 3a	QuantiTect Primer Assay QT01783551	
Dnmt 3b	QuantiTect Primer Assay QT01584625	

### Analysis of PEPCK and DNmt3a2 promoter methylation by pyrosequencing

The level of methylation of individual CpG dinucleotides in the PEPCK promoter was measured in regions between 44 and 658 bp upstream from the transcription start site (Fig. 5A) which had known regulatory function [38], [39]. Four CpG dinucleotides in the Dnmt3a2 promoter was measured in a region 277 bp upstream of the transcription start site [27] (Fig. 5B) by sodium bisulphite pyrosequencing essentially as described [40]. Briefly, genomic DNA was prepared and bisulphite conversion was carried out using the EZ DNA methylation kit (ZymoResearch). The pyrosequencing reaction was carried out using primers listed in Table 3. Modified DNA was amplified using hot startTaq DNA polymerase (Qiagen). PCR products were immobilised on streptavidin–sepharose beads (Amersham), washed, denatured and released into annealing buffer containing the sequencing primers (Table 3). Pyrosequencing was carried out using the SQA kit on a PSQ 96MA machine (Biotage) and the percentage methylation was calculated using the Pyro Q CpG (Biotage). Within assay precision was between 0·8 and 1·8% depending on CpG, and detection limits were 2–5% methylation.

**Table 3:** Pyrosequencing primers.

	Real time RTPCR		
Primer location (bp relative to transcription start site)	Forward Primer (5′<$>\scale 80%\raster="rg1"<$>3′)		Reverse Primer (3′<$>\scale 80%\raster="rg1"<$>5′)
	PEPCK		
	PCR primers		
−658 to −405	AGGGGTTAGTATGTATATAGAGTGATT	ATCAAAACACCACAACTATAAAATATC	
−417 to −56	GTGGTGTTTTGATAATTAGTAGTGATT	CCCCTCAACTAAACCTAAAAACTC	
−373 to −44	GTTAGTAGTATATGAAGTTTAAGA	CCCCTATTAACCAAAAATATATTCC	
−658 to −405	AGGGGTTAGTATGTATATAGAGTGATT	ATCAAAACACCACAACTATAAAATATC	
−417 to −56	GTGGTGTTTTGATAATTAGTAGTGATT	CCCCTCAACTAAACCTAAAAACTC	
−373 to −44	GTTAGTAGTATATGAAGTTTAAGA	CCCCTATTAACCAAAAATATATTCC	
	Sequencing primers		
	GTGATTATTTTATATTAGGTATTG		
	AGAGGATTTAGTAGATATTTAGTG		
	TAAATATTAAAAAACCTCAAACCC		
	TTATTATTTTTTTAAAGTTTATTG		
	Dnmt 3a2		
	PCR primers		
−428 to −63	TTGATGTTTTTTTTTGGTGTGTTT	CAAAAACCTTCAACCCATCAATAA	
−315 to −110	GGTAGGAGGATTGAGAGTTTAGGA	AACAACCCAAACAACTCACCA	
	Sequencing primers		
	GGTTAGAGGATAGATATTGG		
	AGGTTAGAGGATAGATATTG		

### Statistical analysis

Values are shown as mean ± 1 SD. Comparison of single time point data between groups was by 1-way analysis of variance (ANOVA) with maternal generation (F0, F1, F2) or offspring group (CF, F1, F2, F3) as fixed factors, with Bonferroni's *post hoc* test. Measures of changes over time were analysed by ANOVA with time as a repeated measure and maternal generation as a fixed factor with Bonferroni's *post hoc* test. The results of real time RTPCR analysis were non-parametric and were log_10_ transformed before analysis by ANOVA. Analysis of the relationship between PEPCK CpG methylation and mRNA expression was by linear regression.
